# Pins is not required for spindle orientation in the *Drosophila* wing disc

**DOI:** 10.1242/dev.135475

**Published:** 2016-07-15

**Authors:** Dan T. Bergstralh, Holly E. Lovegrove, Izabela Kujawiak, Nicole S. Dawney, Jinwei Zhu, Samantha Cooper, Rongguang Zhang, Daniel St Johnston

**Affiliations:** 1 The Gurdon Institute andthe Department of Genetics, University of Cambridge, Tennis Court Road, Cambridge CB2 1QN, UK; 2 National Center for Protein Science Shanghai, Shanghai Institute of Biological Sciences, Chinese Academy of Sciences, Shanghai 200031, China

**Keywords:** Spindle orientation, *Drosophila*, Mud/NuMA, Pins/LGN, Epithelia

## Abstract

In animal cells, mitotic spindles are oriented by the dynein/dynactin motor complex, which exerts a pulling force on astral microtubules. Dynein/dynactin localization depends on Mud/NUMA, which is typically recruited to the cortex by Pins/LGN. In *Drosophila* neuroblasts, the Inscuteable/Baz/Par-6/aPKC complex recruits Pins apically to induce vertical spindle orientation, whereas in epithelial cells Dlg recruits Pins laterally to orient the spindle horizontally. Here we investigate division orientation in the *Drosophila* imaginal wing disc epithelium. Live imaging reveals that spindle angles vary widely during prometaphase and metaphase, and therefore do not reliably predict division orientation. This finding prompted us to re-examine mutants that have been reported to disrupt division orientation in this tissue. Loss of Mud misorients divisions, but Inscuteable expression and *aPKC*, *dlg* and *pins* mutants have no effect. Furthermore, Mud localizes to the apical-lateral cortex of the wing epithelium independently of both Pins and cell cycle stage. Thus, Pins is not required in the wing disc because there are parallel mechanisms for Mud localization and hence spindle orientation, making it a more robust system than in other epithelia.

## Abstract (precis)

**Highlighted article:** Mud (*Drosophila* NuMA), a crucial spindle orientation factor, does not require its binding partner Pins (*Drosophila* LGN) to localize or function in the *Drosophila* imaginal wing disc.

## INTRODUCTION

Although spindle orientation has been extensively examined in asymmetrically dividing cells, less attention has been given to orientation in symmetrically dividing epithelia. As the tissue develops, most epithelial cell divisions are oriented perpendicular to the plane of the tissue so that both daughter cells lie within the epithelial layer (McCaffrey and Macara, 2011). The orientation of division is determined by the orientation of the mitotic spindle. This orientation depends on a conserved pathway that includes Partner of Inscuteable (Pins; GPR-1/2 in *C. elegans*, LGN or GPSM2 in vertebrates), which anchors Mushroom body defect (Mud; LIN-5 in *C. elegans*, NuMA or NUMA1 in vertebrates) to the cortex. This pathway is thought to work in every mitotic cell type and organism.

To ensure that new cells are born within the plane of the tissue, mitotic spindles must be oriented orthogonally, along the plane. This means that the spindle-orienting machinery must be lateral at mitosis to pull the two spindle poles into alignment. To date, studies into the regulation of this localization have focused on Pins/LGN. Work in Madin-Darby canine kidney (MDCK) cells and in the *Drosophila* imaginal disc has suggested that lateral localization of Pins/LGN is regulated by atypical Protein kinase C (aPKC), which excludes it from the apical cortex (Guilgur et al., 2012; Hao et al., 2010). However, this is not the case in the *Drosophila* follicle epithelium or chick neuroepithelium, in which spindle orientation is aPKC independent (Bergstralh et al., 2013b; Peyre et al., 2011). In these two tissues, the position of the spindle-orienting machinery is determined by the lateral polarity factor Discs large (Dlg), which provides positional information by interacting directly with Pins/LGN (Bergstralh et al., 2013b; Saadaoui et al., 2014). This interaction is mediated by the C-terminal guanylate kinase (GUK) domain in Dlg, which binds a phosphorylated sequence in Pins/LGN (Johnston et al., 2012; Zhu et al., 2011). Binding is thought to be temporally restricted to mitosis by Lgl [L(2)gl – FlyBase], which binds the GUK domain in interphase and is released at mitosis upon phosphorylation by Aurora A/B (Bell et al., 2015; Carvalho et al., 2015). In agreement with this, Lgl variants that cannot be phosphorylated by Aurora A/B remain cortical in mitosis (Bell et al., 2015; Carvalho et al., 2015).

In the current study, we aimed to determine the kinetics of epithelial cell spindle orientation in the *Drosophila* imaginal disc. These measurements led to the unexpected finding that, unlike other well-characterized epithelia, spindle orientation in this tissue proceeds through a Pins-independent mechanism.

## RESULTS

### Spindle angles in the disc vary widely

**Fig. 1.**
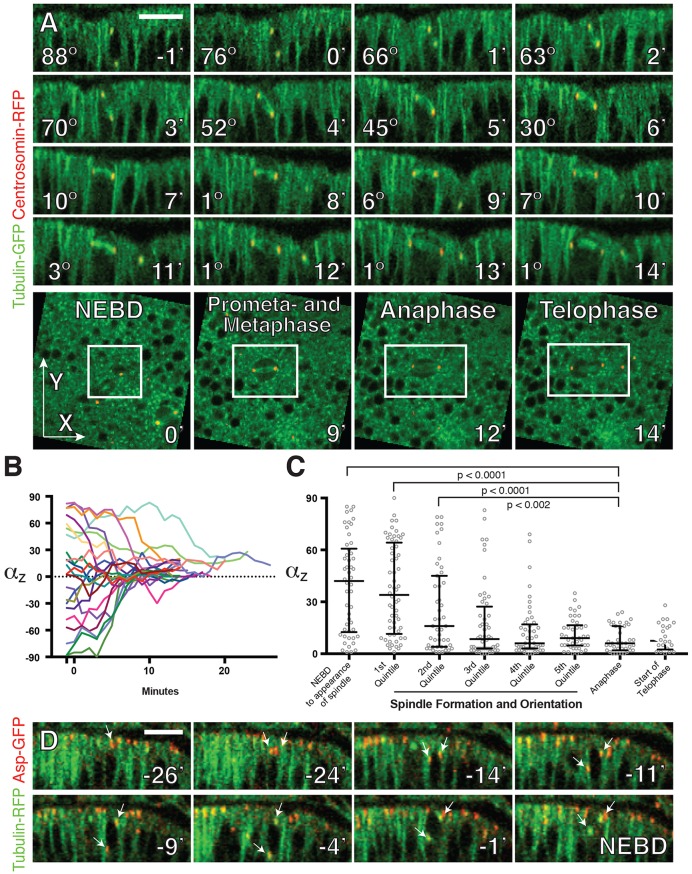
**Mitotic spindle angles vary widely in wild-type *Drosophila* wing discs.** (A) A timecourse showing the development and orientation of the spindle. Timecourse of *z*-reconstructed spindles beginning 1 min before nuclear envelope breakdown (NEBD) (*t*=0) and extending until the appearance of the midbody. Phases of mitosis were confirmed in *xy* (bottom row). The mitotic cell is shown within the boxed regions. The images shown represent five collapsed planes. Centrosomes were marked with Ubi-Cnn-RFP and tubulin with Ubi-α-Tub84B-GFP. (B) Centrosome angles examined over time. Each of the 22 mitoses analyzed (four discs from four flies) was plotted such that the final angle is ≥0°. (C) A comparison of absolute centrosome angles in different phases of mitosis. Anaphase was marked by opposing movement of the centrosomes and by an even distribution of tubulin across the central spindle, in contrast to metaphase where chromosomes exclude tubulin at the center of the spindle. Telophase was marked by the appearance of the midbody. Transition points were all confirmed in *xy* (as in A). The period between the appearance of the spindle and anaphase was normalized as described in the text. Statistical significance was determined using the Kolmogorov-Smirnov test. Bars represent the median and the interquartile distances. (D) Centrosome duplication and movement prior to NEBD. Centrosomes were marked with Ubi-Asp-GFP and tubulin with Ubi-α-Tub-RFP. This represents one of two complete divisions tracked. Arrows point to centrosomes. Scale bars: 10 µm.

We used fluorescently tagged Centrosomin (Cnn) and Tubulin (α-Tub) to follow spindle orientation and cell division in the pouch region of live third larval instar wing imaginal discs. Mitotic angles were determined by drawing a line between the two centrosomes and measuring the angle of this line relative to the tissue plane (these angles are labeled α_z_ in figures). Since this method allowed us to track angles prior to spindle formation, we began our measurements 1 min before nuclear envelope breakdown (NEBD) and continued until the appearance of the midbody, which marked the first minute of telophase (Fig. 1A,B, Movie 1).

We next compared angles at different phases of mitosis (Fig. 1C). Because the time between spindle formation and anaphase varied between divisions, we normalized this period and divided it into quintiles. This resulted in sample sizes and times (mean *n*=50, mean duration=2.3 min) comparable to the period between NEBD and the appearance of a complete spindle (*n*=50, mean duration=2.3 min) and to the period of anaphase (*n*=39, mean duration=1.9 min). Centrosome angles are close to random in the first period (the start of prometaphase) and become oriented an average of 6.9 min later (the third quintile). From this point onwards, the distribution of angles is not statistically significant between groups. Thus, spindles are oriented in the disc roughly halfway between NEBD and anaphase.

Our finding that initial spindle angles are nearly random prompted us to investigate the process of spindle orientation. The starting orientation of the mitotic spindle is anticipated by the positions of the two centrosomes at NEBD. To determine how these positions are established, we followed centrosome duplication and movement over time using Abnormal spindle (Asp)-GFP, which marks centrosomes throughout the cell cycle. We found that the behavior of centrosomes in the wing disc is consistent with previous observations in vertebrate pseudostratified epithelia (Spear and Erickson, 2012; Strzyz et al., 2015; Tsai et al., 2010). During interphase, the centrosome is localized at the apical cell surface. As the nucleus undergoes apically directed interkinetic nuclear migration, the centrosome moves towards the nucleus and divides (Fig. 1D). Although the centrosomes sometimes migrate to equivalent apical-basal positions on either side of the nucleus, in other cases one centrosome remains apical while the other moves basally to the opposite side of the nucleus. This orientation of the centrosomes often persists until the spindle is formed. We considered whether the temporal variability between mitoses could be explained by the time it takes for these vertical spindles to orient, but did not find a correlation between initial spindle angle and division time.

### Divisions orient along the plane of the tissue

We observed a wide variability in spindle angles during the period between NEBD and anaphase, with 58% of spindles having an angle of >30° when they first form and 50% of spindles exceeding this angle for the first half of this period. To determine whether this variability was an artifact of live imaging *ex vivo*, we quantified spindle angles in five wing discs from three dissections. The cumulative distribution of these angles agreed with measurements made in live tissue (Fig. S1A).

The distribution of angles varied between discs, and one fixed disc showed a significantly different mean spindle angle from the cumulative average (Fig. S1A). This reveals that fixed-tissue measurements are very sensitive to the proportion of early spindles in the sample, which can give misleading results. Phospho-histone H3, a standard marker for mitotic cells, cannot be used to exclude these spindles because it appears before the spindle has formed (Fig. S1B). Another potential confounding factor is that one of the centrosomes in an early mitotic cell often lies closer to a centrosome within the same *z*-plane in an adjacent cell than it does to the other centrosome in the same cell, making it difficult to reliably assign spindle angles in the absence of a membrane marker (Fig. S1C).

**Fig. 2.**
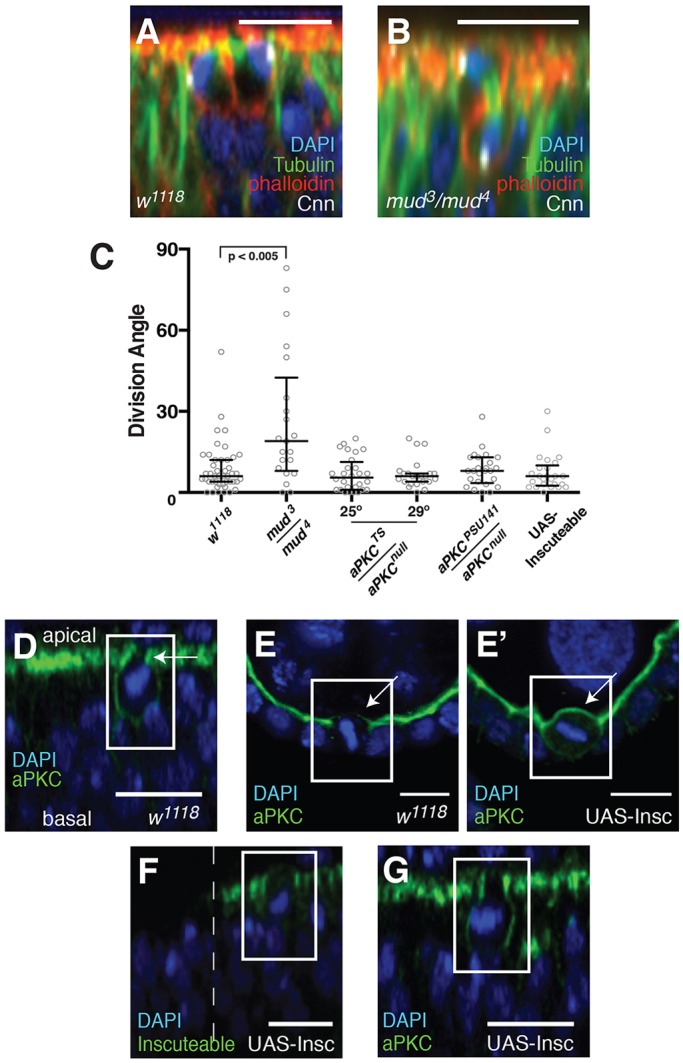
**Division angle is unaffected by mutation of *aPKC* or expression of Inscuteable.** (A) Wild-type wing disc divisions are oriented along the plane of the tissue (*n*=46). (B) A misoriented division in *mud^3^/mud^4^* mutant tissue (*n*=21). (C) The distribution of post-metaphase spindle angles, measured as the angle between the centrosomes and the plane of the tissue, in various mutant conditions. *aPKC^TS^/aPKC^null^*, *n*=27 and *n*=23 at 25°C and 29°C, respectively; *aPKC^PSU141^/aPKC^null^*, *n*=25; UAS-Inscuteable, *n*=25. Statistical significance was determined using the Kolmogorov-Smirnov test. Bars represent the median and the interquartile distances. (D) aPKC (in green) extends downward along the cortex in a mitotic wing disc cell. As at interphase, it is excluded from the apical cortex (arrow). (E,E′) In the follicle epithelium, aPKC is normally lost from the apical cortex at mitosis (E, arrow). It is stabilized by ectopic expression of Inscuteable (E′, arrow). (F) Ectopically expressed Inscuteable, driven by hedgehog-Gal4, localizes to the top of the lateral cortex during both interphase and mitosis in the wing disc. The dashed line indicates the boundary of hedgehog-Gal4 expression. (G) Ectopic expression of Inscuteable in the wing disc does not affect the localization of aPKC in interphase or mitosis. Boxes are drawn around mitotic cells in D-G. Scale bars: 10 µm.

Because of the variability in spindle orientation during metaphase, we restricted our subsequent analysis of division orientation in the wing disc to measurements of post-metaphase cells with separating chromosomes. In agreement with our live imaging measurements, all but one of the angles we measured in fixed tissue was <30° (Fig. S1A). As a positive control for division misorientation, we examined wing discs mutant for the canonical spindle orientation gene *mud* (Fig. 2A-C) (Bell et al., 2015; Kraut et al., 1996; Nakajima et al., 2013; Wodarz et al., 2000, 1999). As expected, the distribution of division angles in these discs differed significantly from the control, confirming that Mud is an essential component of the spindle orientation machinery in these cells (Bergstralh et al., 2013b; Nakajima et al., 2013; Saadaoui et al., 2014).

### The orientation of division does not require aPKC

In MDCK cell cysts, as in most epithelial tissues examined to date, the polarity kinase aPKC localizes along the apical cell cortex, where it has been proposed to play a key role in spindle orientation by phosphorylating LGN (vertebrate Pins) to exclude it from the apical region (Hao et al., 2010). The same mechanism has been implicated in spindle orientation in the *Drosophila* wing imaginal disc (Guilgur et al., 2012). A drawback to this model is that aPKC is not apical in this tissue, but is instead concentrated at the uppermost part of the lateral cortex, suggesting that it is not in the appropriate position to regulate spindle orientation by excluding Pins (Georgiou et al., 2008; Guilgur et al., 2012). One possibility is that aPKC moves apically during mitosis. However, we observed that aPKC spreads down the lateral cortex at mitosis but remains absent from the apical cortex (Fig. 2D). Similar observations have been made in the pupal notum, which derives from the same imaginal disc as the wing (Rosa et al., 2015).

These observations prompted us to re-examine the role of aPKC in the wing disc. Clones of the genetic null allele *aPKC^K06403^* do not survive, but wing discs can be isolated from larvae transheterozygous for *aPKC^K06403^* and the temperature-sensitive allele *aPKC^TS^* (Guilgur et al., 2012; Rolls et al., 2003). Earlier work suggested that spindles are misoriented in these discs at 25°C and higher temperatures (Guilgur et al., 2012). However, the distribution of division angles at anaphase and telophase was normal in discs isolated from these larvae at both 25°C and 29°C (Fig. 2C). Division orientation was also normal in wing discs transheterozygous for *aPKC^K06403^* and the ‘kinase-dead’ allele *aPKC^PSU141^* (Fig. 2C) (Kim et al., 2009). Consistent with reported results using the *aPKC^TS^* allele, extensive apoptotic cell death was observed at the basal surface of these discs (not shown), indicating that aPKC function was compromised (Guilgur et al., 2012). These findings show that aPKC does not regulate spindle orientation in the imaginal wing disc, and are consistent with previous studies in the chick neuroepithelium and the *Drosophila* notum and follicular epithelium (Bergstralh et al., 2013b; Peyre et al., 2011; Rosa et al., 2015). They contrast, however, with work performed in the zebrafish retinal neuroepithelium, since morpholinos targeting aPKCλ/ζ promote division misorientation in that tissue (Cui et al., 2007; Strzyz et al., 2015).

### Ectopically expressed Inscuteable does not reorient divisions in the wing disc

In neuroblasts, apically localized Inscuteable recruits the spindle-orienting machinery to the apical cortex. This provides a pulling force that draws one spindle pole proximal to the apical cortex, thereby aligning the mitotic spindle along the apical-basal axis (reviewed by Bergstralh et al., 2013a). In the *Drosophila* embryonic ectoderm, optic lobe neuroepithelium and follicular epithelium the ectopic expression of Inscuteable performs the same function, resulting in cell divisions that are reoriented by ∼90° relative to the plane of the tissue (Bergstralh et al., 2015; Egger et al., 2007; Kraut et al., 1996). We therefore examined whether Inscuteable also reorients divisions in the wing imaginal disc. Surprisingly, all measured divisions in Inscuteable-expressing wing discs were aligned within 30° of the plane of the epithelium, showing no detectable difference from wild type (Fig. 2C).

We explored the possibility that the failure of Inscuteable to reorient divisions in the wing disc, as it does in other *Drosophila* epithelia, could be attributed to aPKC. In neuroblasts, Inscuteable is recruited to the apical cortex by aPKC. Conversely, Inscuteable is itself required for the apical localization of aPKC (Wodarz et al., 2000). We found that this cooperative localization also occurs when Inscuteable is ectopically expressed in the follicular epithelium. In wild-type mitotic follicle cells, aPKC loses its apical enrichment, spreading out around the cortex (Bergstralh et al., 2013b; Morais-de-Sá and Sunkel, 2013). In mitotic follicle cells expressing Inscuteable, aPKC remains enriched at the apical cortex, although some aPKC also spreads laterally (Fig. 2E). Thus, Inscuteable and aPKC are mutually required to localize apically in this epithelial cell type during mitosis.

This raises the question of whether the same mechanism works in the wing disc, in which aPKC is lateral rather than apical at interphase. We used hedgehog-Gal4 to drive Inscuteable expression in the posterior compartment of the wing pouch. During mitosis, Inscuteable is not apical, as it is in the follicle epithelium, but instead localizes at the top of the lateral cortex (Fig. 2F). The localization of neither aPKC nor its partner Bazooka differs from that in the wild type (Fig. 2G, Fig. S2).

Taken together, these results indicate that Inscuteable can stabilize, but not localize, aPKC at the apical cortex of an epithelial cell. They also provide one possible explanation for why Inscuteable does not reorient spindles in the wing disc: Inscuteable cannot facilitate pulling of just one spindle pole proximal to the apical cortex, since it is not localized apically but in a lateral belt.

### The Dlg/Pins/Lgl pathway does not regulate division orientation in the wing disc

**Fig. 3.**
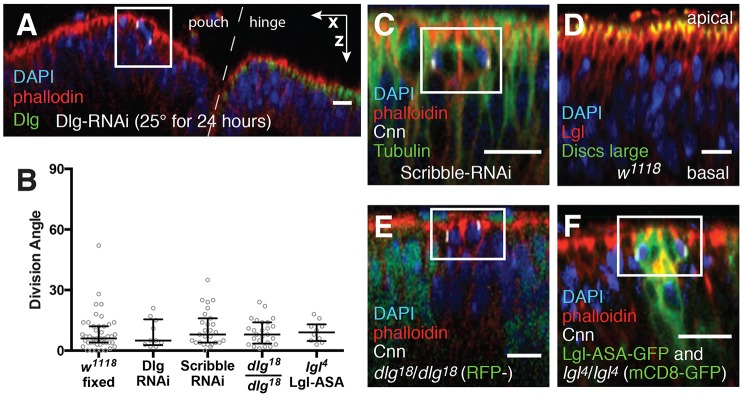
**Spindle orientation in the imaginal wing disc is independent of Dlg and Lgl.** (A) A wing disc from a nubbin-Gal4/UAS-Dlg-shRNAi larvae allowed to develop at 18°C then transferred to 25°C. The organization of the disc is partially maintained. The dashed line illustrates the border between the hinge region and the pouch, in which nubbin-Gal4 is active. A cell dividing along the plane of the tissue is shown in the white box. (B) The distribution of post-metaphase spindle angles in various mutant conditions. Statistical significance was determined using the Kolmogorov-Smirnov test. Bars represent the median and the interquartile distances. Dlg-RNAi, *n*=10; Scribble-RNAi, *n*=27; *dlg^18/18^*, *n*=25; *lgl^4^* Lgl-ASA, *n*=10. (C) A normally oriented division in a Scribble knockdown (nubbin-Gal4/UAS-Scribble-shRNAi) wing pouch. (D) Lgl (red) extends further down the lateral cortex than Dlg (green), which is concentrated in the apical region. (E) Neither tissue organization nor spindle orientation is disrupted in *dlg^18^* wing discs. (F) A normally oriented cell division in an *lgl^4^* clone rescued by expression of Lgl-ASA-GFP. The clone is marked by expression of both mCD8-GFP and Lgl-ASA-GFP. Scale bars: 10 µm.

In the *Drosophila* follicle epithelium and the chick neuroepithelium, Dlg determines the positions of the spindle poles by recruiting Pins to the lateral cortex during mitosis (Bergstralh et al., 2013b; Saadaoui et al., 2014). Previous work identified a spindle orientation defect in wing pouches after Dlg (Dlg1) protein was knocked down using UAS-Dlg-shRNA (TRiP.HMS00024) driven by nubbin-Gal4. In our hands, division angles could not be reliably measured in discs from these larvae grown at the standard temperature (25°C) because the tissue was severely disorganized, as expected from the *dlg* (*dlg1*) mutant phenotype (Fig. S3A) (Gateff, 1978; Nakajima et al., 2013). We could reduce, but not eliminate, disorganization by allowing the tissue to first develop at 18°C to decrease the efficiency of the Gal4 system, then transferring the larvae to 25°C for the last 24 h. Despite loss of Dlg protein (as measured by immunostaining), misoriented divisions were not observed in the more organized regions of these discs (Fig. 3A,B). This suggests that the spindle phenotype observed in Dlg knockdown discs might be due to a loss of epithelial polarity and organization rather than a direct effect on spindle orientation per se. Furthermore, although Dlg has been proposed to act cooperatively with Scribble to orient spindles in the disc, divisions were also oriented normally in UAS-Scribble-shRNA (TRiP.HMS01490) wing discs (Fig. 3C, Fig. S3B).

The role of Lgl in division orientation in the disc is also unclear, since previous work suggests that Lgl is unlikely to protect the Dlg GUK domain from binding to Pins in this tissue. First, the affinity of Dlg for Pins/LGN (*K_D_*=0.33 µM) is over 30 times greater than its affinity for Lgl (*K_D_*=10.2 µM) (Zhu et al., 2011, 2014). Thus, Pins would be expected to simply outcompete Lgl for binding. Second, Dlg is restricted to the top (apical) region of the lateral cortex in the wing disc, whereas Lgl extends further down (Fig. 3D). This suggests that cortical localization of Lgl does not require direct interaction with Dlg, although it does not rule out the possibility that Dlg is required to localize Lgl where they overlap.

These observations prompted us to examine the Dlg/Pins/Lgl pathway directly. In the follicle epithelium, *dlg^18^* is thought to disrupt Pins binding without affecting the essential role that Dlg plays in apical-basal polarity (Bergstralh et al., 2013b). This allele is a nonsense mutation that removes the last 43 residues, comprising roughly one-third of the GUK domain (Woods et al., 1996). This amino acid sequence is highly conserved, suggesting functional importance, but does not include the residues that contact phosphorylated binding partners directly, leaving open the possibility that the truncation does not prevent binding (Fig. S3C). We tested this possibility *in vitro* using vertebrate Dlg4 and LGN (vertebrate Pins). The purified GUK domain lacking the C-terminal 43 residues is unable to bind phosphorylated LGN, its high-affinity target, confirming that the mutation inactivates the phosphoprotein binding activity of the GUK domain (Fig. S3D).

We generated *dlg^18^*/*dlg^18^* mitotic clones in the wing disc and observed that spindle angles within the clones fell within 30° of the plane of the epithelium, as in wild type (Fig. 3B,E) (Bergstralh et al., 2013b). We also found normal localization of Lgl in *dlg^18^*/*dlg^18^* clones, confirming that localization does not require an interaction between single-phosphorylated Lgl and the Dlg GUK domain (Fig. S3E). To test whether the removal of Lgl from the cortex is necessary for correct division orientation in the disc, we expressed Lgl-ASA (S656A, S664A), a variant of the protein that remains cortical during mitosis because it cannot be phosphorylated by the Aurora kinases (Bell et al., 2015; Carvalho et al., 2015). When this construct was expressed in clones of the null allele *lgl^4^* using the MARCM technique, division angles did not differ from wild type (Fig. 3B,F). Division orientation was also normal in *lgl^4^*/*lgl^334^* cells expressing Lgl-ASA (Fig. S3F). Thus, the removal of Lgl from the cortex is not required for correctly aligned divisions in the wing disc. Taken together, these results demonstrate that the Dlg/Pins/Lgl pathway is not required for spindle orientation in this tissue.

### Spindle orientation in the wing disc does not require Pins

**Fig. 4.**
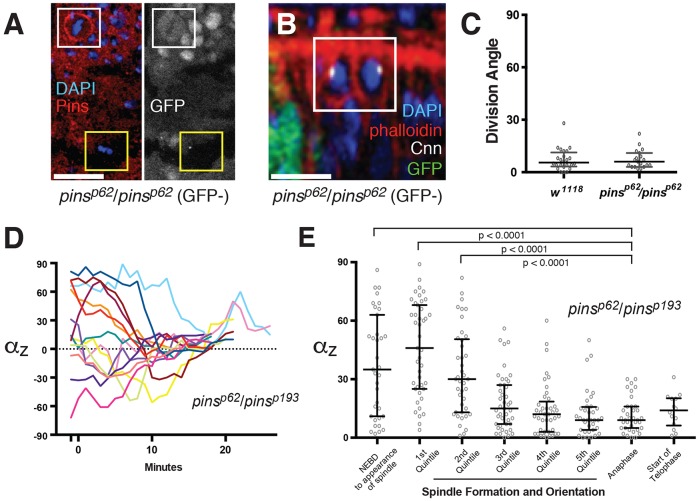
**Spindle orientation is Pins independent in the imaginal wing disc.** (A) In wild-type discs, Pins is cortically enriched during mitosis (white box). It is not detectable in *pins^p62^*/*pins^p62^* tissue (marked by the absence of GFP; yellow box). (B) A normally oriented division in a *pins^p62^*/*pins^p62^* mitotic clone. Mutant tissue is marked by the absence of RFP (green). (C) Quantification of post-metaphase spindle angles in *pins^p62^*/*pins^p62^* tissue (*n*=20). (D) Centrosome angles in *pins^p62^*/*pins^p193^* wing discs examined over time. Each of the 14 mitoses analyzed was plotted such that the final angle is ≥0°. (E) A comparison of absolute centrosome angles in different phases of mitosis. The period between the appearance of the spindle and anaphase was normalized as described for Fig. 1C. Bars represent the median and the interquartile distances. Scale bars: 10 µm.

Pins/LGN is required to orient spindles in every mitotic cell type examined so far, with the exception of the pupal notum (David et al., 2005). However, we have shown that Dlg, Lgl, aPKC and Inscuteable, which are all proposed to exert their spindle-orienting effects through Pins, are not required for division orientation in the wing disc. This raises the question of whether Pins itself is necessary. In agreement with earlier work, we confirmed that Pins is cortically enriched in dividing cells (Fig. 4A, Fig. S4A). To test its functional importance, we generated clones mutant for *pins^p62^*, a 2112 bp deletion that removes the translation start site (Le Borgne and Schweisguth, 2003; Yu et al., 2000). As measured by immunostaining, there is no detectable Pins protein in *pins^p62^* mutant clones, confirming that it is a null allele (Fig. 4A). The *pins^p62^* allele randomizes spindle orientation and misorients divisions in the follicle epithelium (Fig. S4B) (Bergstralh et al., 2013b). By contrast, division orientation is normal in *pins^p62^*/*pins^p62^* wing disc cells (Fig. 4B,C). Thus, the wing imaginal disc epithelium does not require Pins/LGN to orient spindles.

When Dlg is knocked down in the chick neuroepithelium, spindle angles are randomized during metaphase but largely normal at anaphase, suggesting the existence of a correction pathway (Saadaoui et al., 2014). Although Pins is not necessary for spindle orientation in the wing disc, it might still play a redundant role that is compensated for by an anaphase correction mechanism. To address this possibility, we examined spindle orientation throughout mitosis by making time-lapse movies of divisions in wing discs transheterozygous for *pins^p62^* and the strong allele *pins^193^*, a 2658 bp deletion (Parmentier et al., 2000) (Fig. 4C). As we did for the wild type, we normalized progression through mitosis and examined the distribution of centrosome angles at different stages (Fig. 4D). In all of these periods, the distribution of spindle angles in *pins^p62^*/*pins^193^* discs showed no significant deviation from the wild type (Fig. 4E, Fig. S4C). The duration of spindle formation and orientation (appearance of the spindle to anaphase) in *pins^p62^*/*pins^193^* discs was slightly, but not significantly, longer than that of the wild type (control, 12.6±3.3 min; *pins* mutant, 15.5±4.3 min). Thus, Pins is not required for spindle orientation in the wing disc. This experiment also addresses the possibility that aPKC, Inscuteable, Dlg and Lgl spindles undergo correction, since all four factors are thought to mediate spindle orientation through Pins.

### Mud can localize independently of Pins

**Fig. 5.**
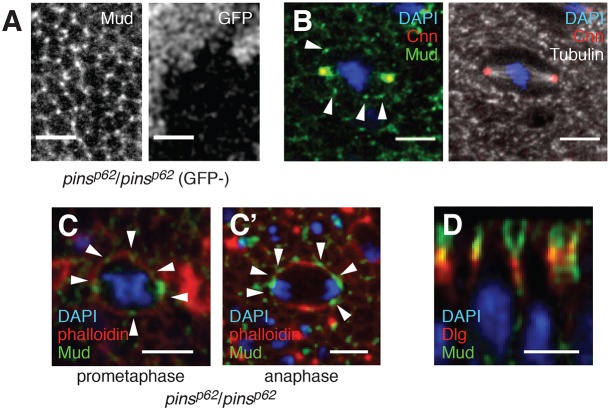
**Mud can localize without Pins.** (A) Mud appears in discrete foci at the interphase cortex. This localization (left panel) is unchanged in *pins^p62^*/*pins^p62^* homozygous clones (marked by the absence of GFP; right panel). (B) The cortical foci persist during mitosis. Additional cortical foci may be proximal to the centrosomes (marked by Cnn) but cannot be distinguished, since Mud is highly enriched. Arrowheads point to the foci. (C,C′) Cortical foci (arrowheads) of Mud are still present in *pins^p62^*/*pins^p62^* mitotic cells at (C) prometaphase and (C′) anaphase. (D) Mud foci extend along the apical portion of the lateral cortex, overlapping with septate junctions (marked by Dlg). This image shows the *x* and *z* planes. Scale bars: 5 µm.

In the canonical pathway, Mud/NuMA orients spindles by exerting a pulling force on astral microtubules. This mechanism is likely to be conserved in the wing disc, since this tissue requires both Mud and centrosomes to orient divisions (Bell et al., 2015; Nakajima et al., 2013; Poulton et al., 2014). However, we have shown that the cortical anchor Pins is dispensable. We therefore examined the localization of Mud in the wing disc. Surprisingly, we observed that Mud localizes to discrete cortical foci, both during interphase and mitosis (Fig. 5A,B). This finding agrees with recently published work (Bosveld et al., 2016). To demonstrate antibody specificity, we used hedgehog-Gal4 to drive expression of UAS-Mud-shRNA (TRiP.HMS01458) in the posterior compartment, which abrogated immunoreactivity (Fig. S4C).

In the disc, Mud localization is not affected in *pins^p62^* mutant clones or in *aPKC^K06403^*/*aPKC^TS^* transheterozygous mutant wing discs (Fig. 5C, Fig. S4E). When viewed along the apical-basal axis, Mud localizes to the apical region of the lateral cortex, where it only partially overlaps with septate junctions (marked by Dlg). These junctions are proximal to spindle poles in this tissue (Nakajima et al., 2013) (Fig. 5D). Thus, Mud is in the appropriate position to orient spindles in the wing disc by a mechanism that is independent of both cell cycle and Pins.

The possibility that Mud can localize without Pins is not without precedent. In cultured vertebrate cells NuMA can associate with the membrane independently of LGN. This depends on the dephosphorylation of NuMA at a conserved Cdk1 target sequence at anaphase (Kiyomitsu and Cheeseman, 2013; Kotak et al., 2013; Seldin et al., 2013; Zheng et al., 2014). Given that the relevant Cdk1 target sequence is not conserved in *Drosophila* (not shown), and that Mud localizes throughout the cell cycle in the wing disc, this mechanism is unlikely to be at work in this tissue. We also explored the possibility that Mud interacts directly with Dishevelled (Dsh), as it does in sensory organ precursor (pI) cells (Ségalen et al., 2010). However, we did not observe defective spindle orientation in *dsh^1^*/*dsh^1^* mutant wing discs (Fig. S4F,G). Thus, the nature of the mechanism that localizes Mud in the wing imaginal disc remains to be identified.

## DISCUSSION

Pins/LGN (GPR-1/2 in nematodes) is required to orient spindles in almost every instance of mitotic spindle orientation in *C. elegans*, *Drosophila* and vertebrate cells studied to date. This includes the symmetrically dividing epithelial cells of the *Drosophila* follicle epithelium and chick neuroepithelium, suggesting that Pins/LGN is broadly required in epithelial tissues (Bergstralh et al., 2013b; Saadaoui et al., 2014). In support of this, we and two other groups have observed cortical localization of Pins in mitotic cells of the *Drosophila* imaginal disc (Dewey et al., 2015; Guilgur et al., 2012). Thus, it seemed likely that Pins retains its spindle orienting function in this tissue. Surprisingly, when we tested the role of Pins directly, we found that it is dispensable for spindle orientation in this tissue. This probably continues into the pupal notum, which derives from the same tissue as the larval imaginal disc (David et al., 2005).

These observations contrast with those of other studies, in which both direct evidence (derived from fixed *pins* mutant tissue) and indirect evidence (derived from fixed tissue lacking the function of putative Pins-regulatory factors) has been used to demonstrate a crucial role for Pins in orienting mitotic spindles in the wing disc. This contradiction is explained by a technical consideration. By imaging spindles in live tissue, we determined that mitotic spindle angles in the wing disc can vary widely over time and are not reliable predictors of division orientation until anaphase. Spindle angle measurements are thus sensitive to the stage of mitosis, which was not accounted for in the earlier work. By restricting our measurements to post-metaphase cell division angles, we determined that neither Pins nor its putative regulatory factors are required to orient divisions in the wing disc.

Pins-independent spindle orientation pathways have been described in three other cases, but none of these seems to be related to spindle orientation in the imaginal wing disc. In sensory organ precursor pI cells in the pupal notum, Mud is recruited to one side of the cell by Pins and to the other side by Dsh (Ségalen et al., 2010). This is unlikely to be relevant, since we have shown that Dsh is dispensable for horizontal orientation in the imaginal disc epithelium. In asymmetrically dividing mouse skin progenitors that divide perpendicularly relative to the plane of the tissue, LGN (vertebrate Pins) cooperates with mInsc (the mammalian ortholog of Inscuteable) to orient spindles along the apical-basal axis (Williams et al., 2014). Surprisingly, LGN is not required for horizontal orientation (Williams et al., 2011). However, this orientation is also independent of NuMA (vertebrate Mud). By contrast, we and others have shown that spindle orientation in the wing disc requires Mud (Nakajima et al., 2013). As mentioned earlier, vertebrate NuMA also has an LGN-independent activity during anaphase (Kiyomitsu and Cheeseman, 2013; Kotak et al., 2013; Seldin et al., 2013; Zheng et al., 2014). This function relies on the dephosphorylation of its C-terminal Cdk1 site, which allows the C-terminal region of the protein to interact with the plasma membrane. However, neither the Cdk1 site nor the C-terminal plasma membrane-binding domain is conserved in *Drosophila*, and the cortical localization of Mud in the wing disc is not cell cycle regulated.

Nevertheless, our data clearly demonstrate that the cortical localization of Mud in the wing disc does not require Pins. In fact, direct examination revealed that even in the absence of Pins, Mud is enriched in cortical foci throughout the cell cycle. This result explains the finding that Pins is not required to orient spindles, but raises further questions about the mechanism that localizes Mud. After submission of our manuscript, another group published that Mud foci in the *Drosophila* notum and wing disc correspond to tricellular junctions (Bosveld et al., 2016). These specialized structures, characterized by distinct protein components including Gliotactin and Anakonda (Bark beetle – FlyBase), form in epithelial tissues with mature septate junctions (Byri et al., 2015; Schulte et al., 2006). Since they are located at lateral cell-cell contacts, they fulfill the requirement for the location of the cortical pulling force that drives horizontal spindle orientation in epithelial tissues.

It is unclear why the wing imaginal disc has evolved a Pins-independent mechanism to orient mitoses. One possibility is that this is related to the fact that spindle orientation is essential to maintain cells in the epithelial layer in this tissue. Misplaced daughter cells in the disc undergo apoptosis and are extruded basally, whereas other epithelia can compensate for misaligned divisions by simply reintegrating the misplaced cells (Bergstralh et al., 2015; Nakajima et al., 2013). These other epithelia have immature septate junctions, and the lateral adhesion proteins that drive reintegration, such as Neuroglian and Fasciclin 2, localize along the entire length of the lateral cortex. In the wing disc, by contrast, the lateral adhesion proteins are tightly restricted to the septate junctions in the apical region of the lateral cortex. This means that they are not in the correct position to adhere to cells that have been basally displaced by misoriented divisions and therefore cannot drive their reintegration. The presence of mature septate junctions in the wing disc also means that this tissue has tricellular junctions, unlike the epithelia in which reintegration occurs. We suggest that in order to compensate for its inability to reintegrate misplaced cells, the wing disc has taken advantage of its tricellular junctions to provide a robust backup mechanism for localizing Mud to the lateral cortex, and thus for spindle orientation.

## MATERIALS AND METHODS

### *Drosophila* mutants

The following mutant alleles and transgenic constructs have been described previously: *mud^3^* and *mud^4^* (Yu et al., 2006), *pins^193^* (Parmentier et al., 2000), *pins^p62^* (Yu et al., 2000), *dlg^18^* (Woods and Bryant, 1989), *aPKC^k06403^* (Wodarz et al., 2000), *apkc^ts^* (*aPKC^TS^*) (Guilgur et al., 2012), *lgl^4^* (Ohshiro et al., 2000), *dsh^1^* (Perrimon and Mahowald, 1987), UAS-Lgl-ASA-GFP (Bell et al., 2015), UAS-Inscuteable (Kraut et al., 1996), nubbin-Gal4 (Thompson and Cohen, 2006) and hedgehog-Gal4 (Tanimoto et al., 2000). *dlg^18^* FRT19A (a gift from Floris Bosveld, Institut Curie, Paris, France) and FRT82B *pins^p62^* were described previously (Bergstralh et al., 2013b). The following background stocks were used to generate mitotic clones, which were induced by heat shock at 37°C for multiple periods of 2 h: *RFP-nls*, *hsflp*, *FRT19A*, *hsflp;FRT40A RFP-nls*, *hsflp;; FRT82B RFP-nls* and *hsflp;; FRT82B GFP-nls*. Mosaic analysis with a repressible cell marker (MARCM; after the method of Lee and Luo, 1999) was carried out using GFP-mCD8 (under the control of an actin promoter) as the marker. The background stock was generated by Aram Sayadian (Gurdon Institute, Cambridge, UK). We thank the Transgenic RNAi Project at Harvard Medical School [NIH/NIGMS R01-GM084947] for providing UAS-Dlg-shRNA (HMS00024), UAS-Scribble-shRNA (HMS01490) and UAS-Mud-shRNA (HMS01458).

### Fluorescent marker stocks

We used the following fluorescent markers: Dlg::YFP (Bergstralh et al., 2013b), Ubi-Asp-GFP (Rujano et al., 2013), Ubi-Cnn-RFP (Basto et al., 2008), Ubi-Cnn-GFP (Conduit et al., 2010), Ubi-α-Tub-RFP (Basto et al., 2008) and Ubi-α-Tub84B-GFP (Rebollo et al., 2004).

### Reagents

The following antibodies were used: rabbit anti-Centrosomin (gift from J. Raff; Lucas and Raff, 2007), rabbit anti-Inscuteable (gift from J. Knoblich; Kraut et al., 1996), rabbit anti-phospho-histone H3 (Ser10, Cell Signaling, #9701, lot #13), rabbit anti-Bazooka (gift from A. Wodarz; Wodarz et al., 2000), rabbit anti-Mud (gift from R. Basto; Rujano et al., 2013), rabbit anti-Pins (gift from F. Matsuzaki; Izumi et al., 2006), rabbit anti-aPKC and anti-Lgl (Santa Cruz, sc-27509, dN-16, lot #H3107), mouse anti-Dlg (Developmental Studies Hybridoma Bank, clone 4F3, 6/5/14), and mouse FITC-conjugated anti-α-Tubulin (Sigma, clone DM1A, lot #114M4817V). Cy5- and Texas Red-conjugated secondary antibodies were purchased from Jackson ImmunoResearch. Phalloidin was purchased from Invitrogen and Vectashield with DAPI was purchased from Vector Labs. Primary and secondary antibodies were used at a dilution of 1:150.

### Imaging

Immunofluorescence and fixed cell imaging were performed as previously described (Bergstralh et al., 2013b). Live imaging was performed using a Leica SP5 (63×/1.4 HCX PL Apo CS oil). *z*-stacks of planes spaced 0.5 µm apart were taken at 1 min intervals. Wing discs were dissected and imaged in 0.8% agarose in Schneider's medium (Sigma) containing 10 µg/ml insulin (Sigma). Images were collected with Leica Application Suite AF and processed (Gaussian blur) using ImageJ (NIH).

### Spindle angle measurements

Centrosome angles were calculated using ImageJ. Angles were determined by drawing a first line connecting the two spindle poles and a second line along the apical surface of the tissue, then measuring the angle between them. These measurements frequently required correction in the *xy* plane, such that both spindle poles were apparent in a single *z*-plane. Statistical analyses were performed using Prism (GraphPad). To prevent biased distribution of fixed images, we counted all angles in each wing disc examined. For analysis of live images, we counted all complete divisions within the 1-h window imaged. Images were analyzed by four independent researchers. As described, centrosome angles were measured from the minute preceding NEBD until the first minute of telophase. NEBD was negatively marked by tubulin, which is clearly excluded from the nucleus prior to NEBD. Telophase was marked by the midbody, which is distinguished by its size, morphology and position.

### Test for correlation

We used the Pearson product-moment correlation to test for correlation between initial spindle angle and the duration of mitosis. We obtained an *r* value of 0.2275, which is less than the critical value of 0.360 for a sample size of 22 (degrees of freedom=20).

### Isothermal titration calorimetry measurements

ITC measurement was performed on an ITC200 microcalorimeter (MicroCal) at 25°C. Protein and peptide samples were dissolved in buffer containing 50 mM Tris pH 8.0, 100 mM NaCl and 1 mM EDTA. The protein concentrations used in the cell (GK mutant) and in the syringe (phospho-LGN peptide) were 0.05 mM and 0.48 mM, respectively. The titration was carried out at time intervals of 2 min to ensure that the titration peak returned to the baseline. The titration data were analyzed using Origin 7.0 software (MicroCal).
